# Combined Preventive Inoculation against Typhoid, Para Typhoids and Dysentery

**Published:** 1916-05

**Authors:** 


**Combined Preventive Inoculation Against Typhoid, Para typhoids and Dysentery.** J. Pratt-Johnson and A. J. Milne. Med. Jour. of S. Africa, Nov., 1915, consider that untreated dysentery vaccine, with or without the other vaccines mentioned, produces too severe and prolonged local reaction. Hence, they use a completely sensitized vaccine, with a heated polyvalent dysentery serum, Shiga, Flexner, Kruse, Hiss and Russell strains being employed in equal parts. This is then combined with the other vaccines. Specific agglutinins are found for each of the diseases, after 9-14 days. 150 inoculations have been made and carefully observed for 14 days, 60% of the persons being engaged in heavy manual labor during the time. They consider their combined vaccine available for military purposes.

